# Ensuring the Future Pool of Gastroenterologists in the United Kingdom Is Imperative

**DOI:** 10.1002/jgh3.70084

**Published:** 2024-12-22

**Authors:** Hareesha Rishab Bharadwaj, Medha Sridhar Rao, Aditya Gaur, Khabab Abbasher Hussien Mohamed Ahmed, Arkadeep Dhali

**Affiliations:** ^1^ Faculty of Biology Medicine and Health The University of Manchester Manchester UK; ^2^ School of Medicine, Dentistry and Biomedical Sciences Queen's University Belfast Belfast UK; ^3^ Yeovil District Hospital Somerset NHS Foundation Trust Yeovil UK; ^4^ Faculty of Medicine University of Khartoum Khartoum Sudan; ^5^ Academic Department of Gastroenterology, Sheffield Teaching Hospitals NHS Foundation Trust Royal Hallamshire Hospital Sheffield UK; ^6^ School of Medicine and Population Health University of Sheffield Sheffield UK

**Keywords:** clinical gastroenterology in the elderly < gastroenterology, gastroenterology, hepatology

## Abstract

This perspective article explores the importance of fostering interest in gastroenterology among medical students and addressing the barriers that deter them from pursuing careers in this specialty. The paper highlights the critical role of early exposure to research, specialized electives, and mentorship in encouraging students to consider gastroenterology as a career choice. Current challenges include limited access to specialized electives and research opportunities within UK medical schools, inadequate hands‐on experience, and a perceived lack of stability and control over future training pathways. Additionally, suboptimal working conditions in the National Health Service (NHS) and uncertainty in the allocation of foundation jobs further discourage students from specializing in gastroenterology. To address these issues, the paper proposes several strategies: expanding gastroenterology electives, increasing research opportunities through grassroots initiatives and developing targeted mentorship programs to guide and inspire students. Furthermore, enhancing diversity and representation within the specialty by removing barriers for female medical students is crucial for creating a more inclusive environment. This article combines insights from existing literature, personal experiences, and innovative educational initiatives to provide a comprehensive overview of the current state of gastroenterology training for medical students. The recommendations aim to inspire new approaches to medical education and training that will cultivate a well‐prepared, diverse, and motivated workforce ready to advance the field of gastroenterology.

**Abbreviations**
AI: artificial intelligence
BSG: British Society of Gastroenterology
CCT: Certificate of Completion of Training
FOBt: fecal occult blood test
IMG: international medical graduate
LLM: large language models
LTFT: less‐than full‐time training
NHS: National Health Service
SMART: specific, measurable, achievable, relevant, time‐bound
UK: United Kingdom

## 1 Introduction

The demand for gastroenterologists in the United Kingdom (UK) is on the rise, driven by an aging population, the expansion of screening programs, and increasing referrals due to national awareness campaigns. The projected need for more gastroenterologists over the next decade is clear, particularly with the planned age extension of the Fecal Occult Blood Test (FOBt) bowel screening program and the introduction of the flexible sigmoidoscopy screening program, which alone is expected to generate approximately 250 000 additional procedures annually. Moreover, the aging population, set to increase by 50% by 2030, is expected to lead to a higher prevalence of gastrointestinal cancers and other age‐related conditions, further increasing the demand for diagnostic and therapeutic gastroenterology services. Furthermore, with a notable proportion of UK gastroenterologists being 55 years or older, there is an anticipated wave of retirements in the coming years, which could further contribute to the need for new specialists to maintain adequate service provision [1, 2].

The pathway to becoming a gastroenterologist in the UK involves completing the Foundation Program after medical school, followed by a three‐year Internal Medicine Specialty Training program. Trainees then enter higher specialty training in gastroenterology, which typically lasts 5 years and includes at least 1 year in a busier hospital and 6 months of training each in specialized liver disease and nutrition. An optional year may be devoted to research. Upon completing this, candidates receive a Certificate of Completion of Training (CCT) and can work as consultant gastroenterologists. Hepatology is the only CCT‐recognized subspecialty, requiring 2 years of liver disease training during specialty training. Other post‐CCT interests include pancreaticobiliary diseases, inflammatory bowel diseases, transplantation, tropical diseases, and clinical nutrition. Some trainees pursue research degrees (MD or PhD) during or before specialty training, although this is less common compared to other academic specialties like neurology [3].

Despite its significant career potential, there has been a gradual decline in interest within the specialty within the UK. Several challenges such as lack of mentorship initiatives, disseminated and sparse research opportunities, barriers to progression, excessive workloads, and lack of support are contributing to staff burnout, discouraging doctors from pursuing the specialty within the NHS [4, 5]. This has also led to a decrease in competition for gastroenterology specialty training and a growing trend of UK graduates seeking opportunities abroad in countries with better working conditions and salaries [6]. Given this backdrop, gastroenterology offers significant career potential for doctors, especially those with dual accreditation in general internal medicine, a necessity for managing complex cases in older populations. It is crucial to encourage more doctors to pursue gastroenterology, ensuring the UK meets the growing demand for specialists. This paper explores strategies for boosting interest in gastroenterology within the UK.

## 2 Strategies to Enhance Interest in Gastroenterology Within the UK

### 2.1 Developing Tailored Electives in Gastroenterology for Medical Students

Gastroenterology offers a rich and diverse learning environment due to the range of acute and chronic conditions it encompasses. Students exposed to clinical settings in gastroenterology gain hands‐on experience in patient care, including pre‐ and post‐procedure evaluations, which strengthens their clinical and procedural skills. Involvement in the field deepens students' understanding of infection control, patient safety, and research application in practice, thus promoting critical thinking [7]. Tailored electives in gastroenterology, focused on the specialty's various aspects, can bridge the gap between theory and practice. By offering structured learning opportunities, students can acquire skills in specialized procedures and develop an understanding of complex cases. These electives should include practical experience, mentorship from seasoned gastroenterologists, and exposure to the latest advancements, from routine assessments to advanced endoscopic interventions. This can inspire students and equip them to tackle the challenges in the field [8].

In the UK, medical students currently benefit from opportunities provided by organizations such as the British Society of Gastroenterology (BSG), as well as elective placements organized by individual NHS trusts and hospitals. However, to better address the growing demand for gastroenterologists and enhance the quality of training, there is a pressing need to expand these opportunities. Innovative approaches could include developing structured, tailored electives that integrate hands‐on experience with cutting‐edge advancements in the field, such as artificial intelligence, digital health, and advanced endoscopic technologies; this could be attained by further collaboration between gastroenterology institutions in the UK and medical schools. Additionally, creating a national platform that centralizes and streamlines information about available opportunities—such as electives, research projects, and mentorship programs—could ensure that all students have equitable access to valuable experiences. This platform could facilitate partnerships between academic institutions, industry, and professional societies, enabling students to engage in interdisciplinary research and innovation from an early stage. Currently, the Royal Society of Medicine offers a similar platform for the opportunities that the society provides.

Furthermore, integrating virtual and remote learning modules could provide additional flexibility and access to specialized training that might not be available locally. Evidence for the benefit of virtual electives has been demonstrated extensively in the current published literature [9]. By expanding and centralizing these opportunities, the platform would not only increase exposure to the specialty but also equip students with the diverse skills and knowledge necessary to tackle emerging challenges and drive future advancements in gastroenterology. Establishing such a comprehensive and accessible system would foster a new generation of gastroenterologists prepared to contribute to the evolving landscape of the field, ultimately addressing workforce shortages and improving patient care through a more robust and innovative training framework.

### 2.2 Fostering Gastroenterology Research Engagement at the Grassroots Level in Medical School

Fostering gastroenterology research in medical school is crucial for several reasons, including advancing scientific knowledge, improving patient care, and developing the next generation of specialists. Research engagement at the medical school level helps bridge the gap between theoretical learning and practical application, allowing students to apply their knowledge to real‐world problems and contribute to innovative solutions [10]. Early exposure to research fosters critical thinking, problem‐solving skills, and a deeper understanding of disease mechanisms, which are essential for diagnosing and treating complex gastroenterological conditions. Moreover, engaging students in research can ignite a passion for gastroenterology by showcasing the specialty's dynamic and impactful nature. Students who participate in research are more likely to appreciate the field's complexity and relevance, which can lead to a stronger interest in pursuing gastroenterology as a career. This involvement provides a tangible connection to the specialty's cutting‐edge advancements and challenges, making it a compelling choice for those drawn to both clinical practice and scientific discovery [10, 11]. As students explore research opportunities, they gain insights into the scientific process, from hypothesis formulation to data analysis and interpretation. This experience not only enhances their clinical skills but also prepares them for potential careers in research or academia, where they can continue to advance the field of gastroenterology.

Undergraduate research societies play a pivotal role in fostering gastroenterology research among medical students by creating a supportive environment for early involvement in scientific inquiry. These societies not only provide platforms for students to engage with research but also cultivate interest in specialized fields like gastroenterology through innovative and targeted initiatives [11, 12]. Consequently, there is a need for a greater expansion of undergraduate student societies focused on research in gastroenterology in UK medical schools. A noteworthy grassroots initiative in the UK is The MedAhead Society's “ResearchRevive” program, which exemplifies how well‐structured educational interventions can significantly enhance research engagement among medical students, particularly those from widening participation backgrounds. This initiative offers a robust online curriculum covering research methodologies such as study design, data collection, statistical analysis, and ethical considerations. By addressing notable deficiencies in formal research education within UK medical schools, “ResearchRevive” provides students with crucial research skills and cultivates a supportive learning environment that promotes active participation and ongoing professional development [13].

One innovative approach is the development of research‐focused seminars and workshops specifically tailored to gastroenterology. These can include hands‐on sessions with advanced diagnostic technologies and experimental techniques that are currently shaping the field. By integrating these practical experiences with theoretical knowledge, students gain a deeper understanding of the field and its evolving landscape. Another valuable initiative is establishing collaborative research projects between undergraduate research societies and specialized gastroenterology departments. This can involve mentoring programs where experienced researchers guide students through the process of designing, executing, and analyzing research projects. Such collaborations can lead to joint publications and presentations, providing students with a tangible sense of achievement and a clearer pathway into the field. Virtual research forums and online communities dedicated to gastroenterology can also be a significant asset. These platforms can facilitate discussions, share recent developments, and connect students with experts globally, offering a broader perspective on the specialty. They can host webinars, virtual lab tours, and interactive case studies that engage students in current research challenges and innovations. Additionally, tying into the previously discussed endeavor, undergraduate research societies can advocate for and organize specialized elective courses or mini‐internships within gastroenterology departments. These opportunities would allow students to immerse themselves in the field, participate in ongoing research projects, and experience the day‐to‐day work of gastroenterologists and researchers.

### 2.3 Developing Gastroenterology Mentorship Initiatives for Medical Students

Developing gastroenterology‐focused mentorship programs provides medical students with direct guidance in clinical practice and portfolio development. These programs would cover various aspects such as clinical knowledge, research, and offer a mentor's deeper insight into the practice of gastroenterology and the pathway to specialized training [14]. A critical consideration is the mentor‐to‐mentee ratio, as it can significantly affect the mentee's overall experience. Effective mentorship often depends on one‐on‐one relationships and the mentee's proactive engagement. A key strategy for mentees is to set realistic and specific goals, using the SMART criteria (Specific, Measurable, Achievable, Relevant, Time‐bound). Establishing both short‐term and long‐term objectives at the beginning of the program serves as a roadmap for the mentor‐mentee pair throughout the mentorship [14, 15]. Successful mentorship programs have demonstrated positive outcomes, including improved technical skills, enhanced career guidance, and valuable insights into training opportunities [10]. Immediate benefits have also been observed, such as better academic performance and increased research productivity [15].

Mentorship initiatives in medical schools can be organized by national bodies or through gastroenterology departments within NHS trusts affiliated with medical schools. For instance, the BSG launched its mentorship scheme in late 2022, matching mentors and mentees based on shared interests and goals [16]. This matching process facilitates a more holistic mentorship experience, allowing mentors to share their expertise in specific areas and research while mentees gain valuable insights into specialized gastroenterology topics, thus enhancing their passion and understanding of the field. The duration of mentorship schemes is also an important consideration, as both long‐term and short‐term programs offer distinct advantages and challenges. Long‐term mentorships provide opportunities for extended projects, such as audits and quality improvement studies, fostering enduring mentor‐mentee relationships. Conversely, short‐term programs allow mentors to work with a variety of mentees, giving students the chance to learn from different mentors and gain diverse perspectives on gastroenterology. Determining the optimal duration for mentorship schemes can be guided by evaluating the student's current stage in medical school or level of interest in gastroenterology. For example, students in earlier years might benefit more from short‐term programs to explore the specialty, whereas those in clinical years or with a strong interest in gastroenterology may prefer long‐term mentorships. Longer‐term schemes often create environments where mentees are inspired to become mentors themselves, experiencing greater career progression [15, 16, 17, 18].

### 2.4 Improving Equality, Diversity and Inclusion Within UK Gastroenterology

Inequalities in UK gastroenterology remain pervasive, particularly regarding gender, ethnicity, and professional background. While women comprise over 60% of medical students, they account for only 38.6% of higher specialist trainees and 22.3% of gastroenterology consultants. Similarly, people of color, despite representing 45.3% of trainees, constitute just 32.9% of consultants, with women of color being the most underrepresented group. These disparities highlight systemic issues, including unequal access to leadership roles, lack of mentorship, and biases in recruitment and promotion processes. The absence of meaningful diversity targets and accountability mechanisms exacerbates these issues, perpetuating inequities across the specialty. Additionally, International Medical Graduates (IMGs), who account for over 52% of newly registered doctors, encounter significant challenges such as complex visa processes, limited support during NHS transitions, and difficulties in accessing specialist training. These barriers often result in the underutilization of their skills, compounding workforce gaps in gastroenterology [19, 20, 21].

To address these challenges, gastroenterology programs must adopt targeted strategies to foster equality, diversity, and inclusion. Flexible training models, such as less‐than‐full‐time (LTFT) options, can ensure that individuals balancing caregiving responsibilities or other personal challenges are not disadvantaged. Standardized return‐to‐work programs, including refresher courses and reorientation sessions, should be implemented across NHS trusts to support individuals returning from extended leave, such as maternity or health‐related absences. Similarly, institutions must safeguard trainees from occupational risks like radiation exposure during pregnancy by offering alternative responsibilities. For IMGs, tailored induction programs, expedited visa processes, and mentorship initiatives pairing them with experienced clinicians can facilitate smoother transitions to NHS practice and foster their professional growth.

Promoting diversity in leadership and academic roles is equally critical. Mentorship initiatives and peer‐support networks designed specifically for underrepresented groups, including IMGs, can provide essential guidance and reduce professional isolation. Visible role models from diverse backgrounds—highlighted through conferences, research projects, and academic forums—can inspire future generations of gastroenterologists. Structured bridging programs for IMGs to address qualification gaps and enable access to specialist training can further strengthen their contribution to the workforce.

Setting measurable diversity targets is essential for accountability. UK gastroenterology organizations should emulate initiatives like the American Gastroenterological Association Equity Project by introducing minimum diversity thresholds for speaker panels and leadership positions. For example, ensuring at least one‐third of speakers at academic events are women or people of color can normalize diverse representation. Committees dedicated to monitoring gender, ethnicity, and broader diversity metrics should evaluate progress through annual reports and revise strategies every 3–4 years to reflect evolving workforce demographics. Addressing systemic inequalities also requires equitable access to research funding and fellowship opportunities. Historically, male applicants and those from majority ethnic groups have enjoyed greater success in securing these opportunities. Funding programs targeted at underrepresented groups, alongside workshops on grant writing and leadership development, can empower marginalized trainees to overcome these barriers.

Creating a culture of inclusion within gastroenterology is paramount. Embedding equality, diversity and inclusion principles into the framework of medical societies, NHS trusts, and training programs can ensure that leadership reflects the workforce's diversity. By fostering an environment where all professionals can thrive, gastroenterology will be better equipped to serve patients, innovate, and address workforce challenges. A bold commitment to equity will benefit not only underrepresented groups but also the specialty and the broader healthcare system [19, 21].

### 2.5 Leveraging Artificial Intelligence and Large Language Models in Gastroenterology

The integration of artificial intelligence (AI) and large language models (LLMs) into gastroenterology presents a transformative opportunity to enhance clinical efficiency, improve diagnostic precision, and support patient‐centered care. AI‐powered tools, such as advanced systems for polyp detection during endoscopy, have demonstrated their ability to enhance diagnostic accuracy, reduce interobserver variability, and streamline procedural workflows. Similarly, LLMs can assist clinicians by automating administrative tasks, such as summarizing patient records, drafting reports, and personalizing patient education materials, freeing up valuable time for direct patient interactions and complex clinical decision‐making [22].

Despite these benefits, the widespread adoption of AI and LLMs faces significant barriers. Concerns about data privacy, potential algorithmic biases, and the lack of AI literacy among healthcare professionals remain pressing challenges. Without proper understanding, clinicians may struggle to utilize these technologies effectively, risking suboptimal implementation and missed opportunities to improve care.

To overcome these challenges, fostering AI literacy among gastroenterologists is crucial. National and international initiatives, such as the British Society of Gastroenterology's AI Task Force, play a pivotal role in addressing this gap. These programs provide structured training to equip clinicians with the skills to understand, interpret, and safely apply AI technologies in their practice. Alongside educational efforts, the establishment of robust governance frameworks is essential to ensure ethical AI deployment. Transparency in algorithmic processes, safeguards to mitigate biases, and a focus on patient safety must underpin AI integration in clinical settings.

Moreover, collaboration across disciplines—spanning healthcare providers, technologists, and policymakers—is fundamental to ensuring that AI adoption is both clinically effective and equitable. Such efforts can drive the responsible implementation of AI, unlocking its potential to address workforce shortages, reduce workload pressures, and deliver sustainable, high‐quality care in gastroenterology. By embracing these innovations, the gastroenterology community can transform its practice, ensuring it remains at the forefront of technological advancements while prioritizing patient outcomes [22, 23].

## 3 Conclusion

In conclusion, prompt action is needed to improve the interest in gastroenterology among medical students to address the anticipated shortage of gastroenterologists. To this end, expanding gastroenterology electives, increasing research opportunities through grassroots initiatives and developing targeted mentorship programs to guide and inspire students are imperative. These improvements, coupled with robust mentorship and professional development opportunities, will help attract and retain talented individuals, ensuring a well‐supported workforce capable of meeting the growing demand for gastroenterologists. Ultimately, these efforts are essential for building a resilient gastroenterology sector within the UK healthcare system.

## Ethics Statement

The authors have nothing to report.

## Conflicts of Interest

The authors declare no conflicts of interest.

## Data Availability

Data sharing is not applicable to this article, as no new data were created or analyzed in this study.
